# Lordoscoliosis and hyperlordosis in quadriplegic cerebral palsy

**DOI:** 10.11604/pamj.2020.36.242.24971

**Published:** 2020-08-04

**Authors:** Chanan Vivek Goyal, Waqar Mohsin Naqvi

**Affiliations:** 1 Government Physiotherapy College, Raipur, Chhattisgarh, India,; 2 Community Health Physiotherapy Department, Ravi Nair Physiotherapy College, Datta Meghe Institute of Medical Sciences, Wardha, Maharashtra, India

**Keywords:** Cerebral palsy, lordoscoliosis, pediatrics deformities, hyperlordosis, posture

## Image in medicine

A 7-year-old female, born out of a non-consanguineous marriage, presented with an extreme posturing of the spine. As per her father, she had a history of delayed birth cry and recurrent episodes of seizures. She was diagnosed with quadriplegic cerebral palsy with epilepsy. She was referred by an orthopedic surgeon to the department of physiotherapy for evaluating if the spinal deformity was fixed or flexible, so that the further line of management could be determined. On observation of her posture in supine lying (A) and in prone lying (B), marked lordoscolios was noted along with hyperlordosis. Examination revealed spasticity of grade 3 on the modified Ashworth scale (MAS) in spinal extensor muscle group. She was neither able to roll over nor was she able to sit even with extensive support. The posturing of the spine posed difficulties for caregivers during feeding, dressing and during activities to maintain hygiene. In an attempt to examine flexibility of the spine, child was placed prone on the lap by the physiotherapist. Slow vestibular stimulus in the form of rocking movements was given by the movement of the physiotherapist´s thighs. As the child displayed some relaxation of trunk muscles, the physiotherapist clasped her hands below her knees providing a firm pressure to the child´s back through her arms. This further inhibited the resistance offered by the child´s muscles and the spine could be aligned to near neutral in the sagittal as well as in the coronal plane revealing the flexible nature of the deformity (C).

**Figure 1:**
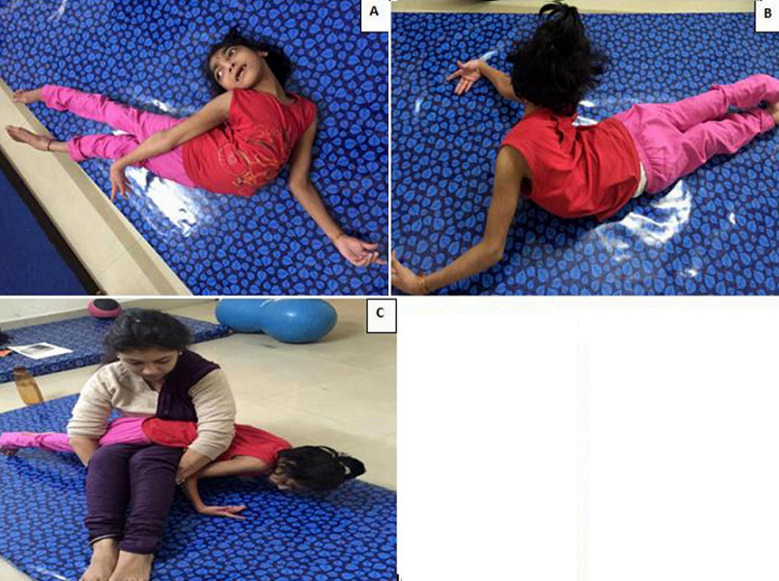
A) posture in supine lying; B) in prone lying, marked lordoscoliosis along with hyperhidrosis; C) is showing the spine alignment to near neutral in the planes revealing the flexible nature of the deformity

